# Lipids, Lipoproteins, and Age-Related Macular Degeneration

**DOI:** 10.1155/2011/802059

**Published:** 2011-07-28

**Authors:** Katayoon B. Ebrahimi, James T. Handa

**Affiliations:** Retina Division, Wilmer Eye Institute, Johns Hopkins School of Medicine, Baltimore, MD 21287, USA

## Abstract

Age-related macular degeneration (AMD) is the leading cause of blindness among the elderly. While excellent treatment has emerged for neovascular disease, treatment for early AMD is lacking due to an incomplete understanding of the early molecular events. A prominent age-related change is the accumulation of neutral lipid in normal Bruch's membrane (BrM) throughout adulthood and also disease-related BrM accumulations called basal deposits and drusen. AMD lesion formation has thus been conceptualized as sharing mechanisms with atherosclerotic plaque formation, where low-density lipoprotein (LDL) retention within the arterial wall initiates a cascade of pathologic events. However, we do not yet understand how lipoproteins contribute to AMD. This paper explores how systemic and local production of lipoproteins might contribute to the pathogenesis of AMD.

## 1. Introduction

Age-related macular degeneration (AMD) is the leading cause of blindness among the elderly in the United States, representing 54% of legal blindness [1]. Currently, 1.75 million people are affected by advanced AMD, and due to the aging population, 3 million people will be affected by 2020 [2]. At present, 7 million people are at risk of developing advanced AMD, and 1 in 3 persons ≥70 years old with early AMD will develop advanced disease over 10 years [1, 3]. The devastating impact to both the individual and general public is staggering. In Canada and Australia, the financial impact is estimated to be $2.6 billion on the gross domestic product [4, 5]. The continued trend for increased life expectancy predicts a doubling in the number of people with AMD with costs reaching $59 billion over the next 20 years [5]. Improving treatments that reverse, prevent, or even delay the onset of AMD would have significant benefit to both the individual and society. 

A growing body of the literature indicates the involvement of lipids and lipoproteins in the formation of extracellular lesions in aging Bruch's membrane, basal deposits and drusen, and two hallmark AMD lesions. In this paper, we will explore the various aspects for how lipoprotein particles might contribute to the pathogenesis of AMD.

## 2. Anatomy and Histology of the Macula

**Figure 1:**
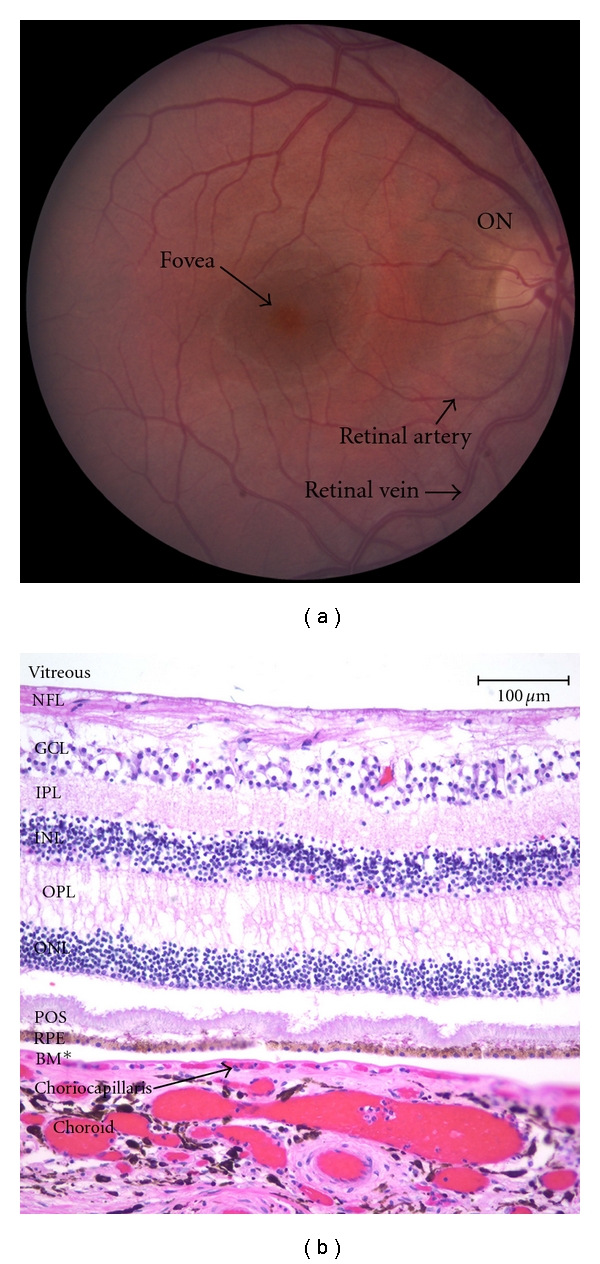
(a) Fundus photo of the right eye. The macula is located between vascular arcades with the fovea at the center approximately 3 mm temporal to the optic nerve (ON). (b) Histopathological section of the neurosensory retina from the inner (vitreous) to outer layers: nerve fiber layer (NFL), ganglion cell layer (GCL), inner plexiform layer, inner nuclear layer (INL), outer plexiform layer (OPL), outer nuclear layer (ONL), outer segments of the photoreceptors (POS), underlying retinal pigment epithelium (RPE), Bruch's membrane (BM*), and choriocapillaris and choroid (Hematoxylin-eosin).

The retina arises from the optic vesicle and consists of a retinal pigment epithelial (RPE) layer, which is derived from the optic cup, and a complex sensory layer, which is derived from the inner layer of neuroectodermal cells of the optic cup [6]. The sensory retina is transparent, and adjacent to the RPE, is composed of nine layers: the rod and cone photoreceptors, the external limiting membrane, the outer nuclear layer, the outer plexiform layer, the inner nuclear layer which contains the nuclei of horizontal cells, bipolar cells, amacrine cells, and Muller cells, the inner plexiform layer, the ganglion cell layer, the nerve fiber layer, and the internal limiting membrane, which is next to the vitreous gel (Figure 1(b)). While the peripheral retina and much of the macula, or the central retina, is dominated by rods in a 20 : 1 and 9 : 1 ratio, respectively, the central-most aspect of the macula, called the foveola, is comprised exclusively of cones [6–8]. This enriched area of cones and their close packing accounts for the high visual acuity.

Clinically, the macula is defined as the central retina that is located within the vascular arcades (Figure 1(a)). Histologically, it is the region that contains more than 1 layer of ganglion cell nuclei [9]. The term macula lutea is derived from the yellow color of the central retina which is due to carotenoid pigments within the Henle fiber layer. Two of the major yellow pigments that have been identified are zeaxanthin and lutein. The former is concentrated in cone dense areas while lutein is concentrated in rod dense areas of retina. The fovea is a concave central depression 1.5 mm in diameter and is seen on clinical examination as an elliptical light reflex that arises from the slope of the thickened internal limiting of the retina. The foveola is a central 0.35 mm diameter depression within the fovea. Usually, only cone photoreceptors and Muller cells are present in this area [6]. 

The inner retina derives its nutrition from the retinal vascular system while the outer layers, including the outer portion of the inner nuclear layer, derive their nutrition from the choriocapillaris. The central 250 to 600 *μ*m of the macula or foveal avascular zone is free of retinal blood vessels and derives its nutrition from the choroid [10, 11].

The neurosensory retina is supported by the retinal pigmented epithelium (RPE), the polarized epithelial monolayer. Like the photoreceptors and outer retinal layers, the RPE relies on the choroid for its nutrition [12]. The 200–300 mm thick choroid has the highest blood flow per unit tissue perfused in the body, with 7-fold greater flow in the macula relative to the periphery [13]. The RPE plays an important role in vitamin A metabolism, outer blood-retina barrier integrity, daily phagocytosis of the photoreceptor outer segments, absorption of light, heat exchange, formation of the basal lamina, production of the mucopolysaccharide matrix surrounding the outer segments, secretion of factors that maintain the choriocapillaris, the polarized secretion of cytokines, and active transport of materials in and out of the RPE. In the adult eye, the RPE is mitotically inactive [14]. 

The thin Bruch's membrane (BrM) is a pentalaminar matrix located between RPE and choroid. BrM is a semipermeable filtration barrier through which major metabolic exchange takes place. Ultrastructurally, it has 5 layers: the RPE basement membrane, the inner collagenous zone, the elastic zone, the outer collagenous zone, and the choriocapillaris basement membrane [15]. The interfiber matrix of BrM is largely composed of heparin sulfate and chondroitin/dermatan sulfate, and it has been suggested that the chondroitin sulfate side chains provide an electrolytic barrier to diffusion. Maximum diffusion occurs at the membrane isoelectric point of pH 5. At physiologic pH, there is a negative charge, and this may impede the passage of negatively charged macromolecules. Any alteration in the structure or composition of BrM might influence its diffusion properties and ultimately the function of the RPE and outer retina [16–19].

## 3. What Is Age-Related Macular Degeneration (AMD)?

**Figure 2:**
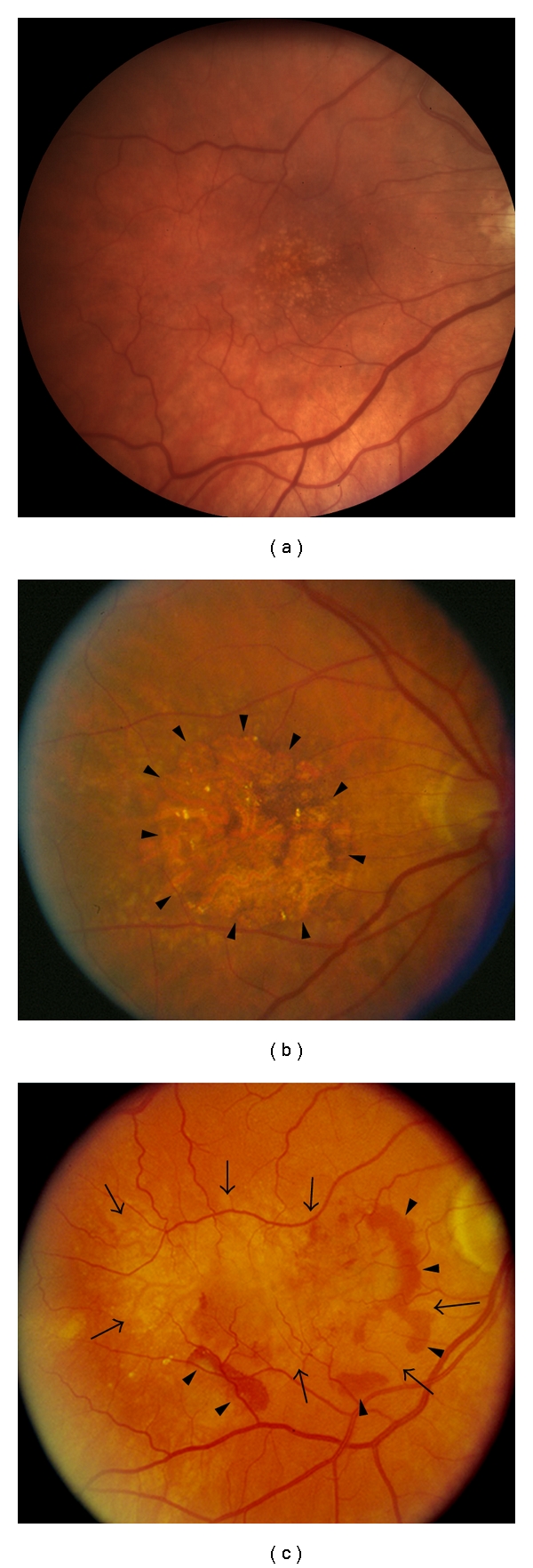
(a) Fundus photo of the right eye with early non-neovascular (dry) AMD. Note the numerous yellow subretinal deposits (Drusen). (b) Fundus photo of the right eye with advanced nonneovascular AMD (Geographic atrophy). Note area where RPE cells have died from apoptosis (arrowheads). (c) Fundus photo of the right eye with neovascular or wet AMD. Note subretinal hemorrhage (arrowheads) adjacent to a choroidal neovascular membrane (arrows).

AMD is typically classified into two clinical forms, “dry” or “wet” (Figure 2), both of which can lead to visual loss. In the dry or nonneovascular form, visual loss is usually gradual. The hallmark changes that are seen in the macula are yellow subretinal deposits called drusen, or RPE hyperpigmentary or hypopigmentary irregularities. Drusen may enlarge and become confluent, and even evolve into drusenoid RPE detachments, where the RPE becomes separated from its underlying Bruch's membrane. These drusenoid detachments often progress to geographic atrophy, where the RPE dies from apoptosis or into wet AMD. In the wet form, also called exudative or neovascular AMD, vision loss can occur suddenly when a choroidal neovascular membrane develops in the sub-RPE, between the RPE and Bruch's membrane, and it subretinal space, between the neurosensory retina and RPE, and it leaks fluid or blood [16].

At present, the pathophysiology of AMD is incompletely understood. AMD is a complex trait genetic disease modulated by various nongenetic or “environmental” risk factors [20]. Nonmodifiable risk factors include increasing age, gender, and family history [20, 21]. To date, chronological aging remains the strongest risk factor. A number of other risk factors have also been reported to influence AMD, including smoking, diet, higher body mass index, serum cholesterol levels, cataract surgery, cardiovascular disease, hypertension, and sunlight exposure [20–22]. Among these factors, cigarette smoking is the strongest risk factor [23]. A “dose-response” effect has been established since pack-year smoking strongly correlates with AMD while smoking cessation reduces the risk for AMD [24]. Epidemiologic data from several large studies indicate that both AMD and disease progression are strongly influenced by smoking [22–26].

## 4. Histopathology of AMD

### 4.1. The RPE and Aging

The RPE is highly metabolically active due to a number of specialized functions that sustain photoreceptor health. With advancing age, RPE cells undergo an increase in pleomorphism, decrease in melanin content, and an overall decrease in cell number within the posterior pole [27]. For this study, we will focus our discussion on photoreceptor processing because (a) photoreceptor outer segments (POSs) are lipid rich and (b) of the magnitude of POS processing by the RPE cell. Throughout life, RPE cells utilize tremendous energy to phagocytose and recycle lipid-rich POS back to the photoreceptors in order to preserve visual function. In fact, the RPE cycles approximately 30,000 lipid-rich POS per day [28]. Disruption of this recycling over time can result in RPE and photoreceptor dysfunction in part, due to the accumulation of lipid and lipid peroxidation products [29]. Engulfed POSs are contained in phagosomes which are processed in lysosomes and broken down into their individual components for recycling. Aging reduces the efficiency of lysosomal processing and results in the accumulation of incompletely digested phospholipids [30].

The accumulation of cellular lipid does not appear to have a deleterious impact upon RPE function. However, lipid accumulation, combined with oxidative stress over time, results in the formation of lipid peroxidation products, such as 4-hydroxynonenal (HNE) and malondialdehyde (MDA) [31]. Suzuki et al. found, within macular photoreceptors and the RPE of normal eyes, increasing quantities of oxidized phospholipids with advancing age [32]. Mechanistically, these lipid peroxidation products prevent POS proteins from lysosomal degradation [31]. The undigested end products within the phagolysosome, or residual bodies, accumulate with aging. Progressive accumulation of the undigested lipid peroxidation products will stress the RPE, which can ultimately induce apoptosis, a well-established process in aging and AMD, unless they are removed from the cell by exocytosis, transcytosis, or autophagy [33–37]. These changes can influence Bruch's membrane. The released particles including apoptotic particles into Bruch's membrane can become the inflammatory nidus for basal deposit and drusen formation [13, 16, 38–43]. The impact of lipid peroxidation product processing on the photoreceptors, RPE, and Bruch's membrane remains a relatively understudied area. Given the potential impact on AMD, it appears to be a fruitful avenue for future study.

### 4.2. Aging Bruch's Membrane, Basal Deposits, and Drusen

**Figure 3:**
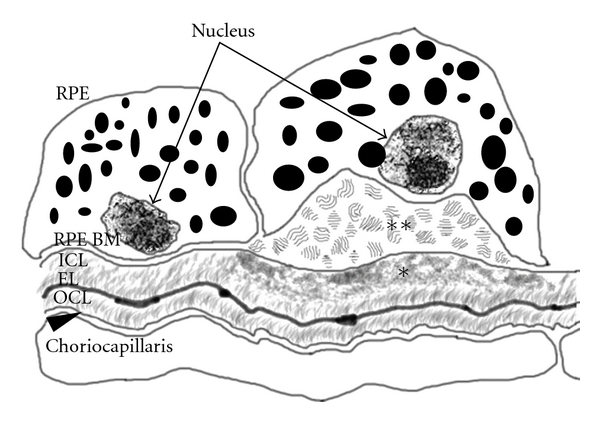
Schematic image of the RPE-Bruch's membrane-choriocapillaris interface in AMD. Basal laminar deposits (BlamDs; **) appear between the RPE cell and the RPE basement membrane (RPE BM), while basal linear deposits (BlinDs; *) localize at the inner collagenous layer (ICL) beneath the RPE basement membrane. EL: elastin layer; OCL: outer collagenous layer; arrowhead indicates endothelial cell basement membrane.

With chronological aging, Bruch's membrane thickens and develops heterogeneous deposits called basal deposits. The location and composition of basal deposits help to distinguish aging from AMD changes. The earliest age-related deposits are seen within the outer collagenous layer, and occur as early as 19 years of age [44]. These outer layer deposits are the main contributors to the generalized age-related thickening of Bruch's membrane. Deposits between the RPE cell and its basement membrane, known as basal laminar deposits or BlamD (Figure 3), are also an age-related change when thin and homogeneous in composition. Early lesions look identical to the RPE basement membrane on electron microscopy and consist, in part, of normal RPE basement membrane molecules such as collagen IV, VI, laminin, and heparan sulfate proteoglycans, which seem to result from either excess production or reduced degradation[44–49]. Other proteins such as vitronectin, MMP-7, TIMP-3, C3, and C5b-9 are found in both BlamD and drusen [13, 50]. When BlamDs thicken and accumulate heterogeneous debris, “long spacing collagen,” lipoproteins, and inflammatory proteins, they are associated histopathologically with AMD. When deposits accumulate within the inner collagenous layer, they are designated as basal linear deposits, or BlinDs (Figure 3), which is one of the strongest histopathological markers for AMD. When sufficient debris, including lipid, accumulates within BlinD to form a mound, it can be seen clinically as large drusen (>125 *μ*m) [49, 51, 52].

### 4.3. Histopathologic Evidence of Intraocular Lipid Particles within Bruch's Membrane

Lipid particles accumulate within Bruch's membrane in the exact location and prior to the development of basal deposits or drusen. This observation has led to the hypothesis that these lipid particles contribute to drusen formation during the development of AMD. 

Sarks et al. [53] first described these lipid particles as membranous debris within BrM, basal deposits, and drusen within the macula. Membranous debris is visualized by transmission electron microscopy (TEM) and appear as irregularly shaped and sized particles (range 33 to 267 nm diameter) with empty interiors [51]. The varying size of membranous debris may result from aggregations of particles, variable loading of lipids into lipoprotein particles, variable degrees of extracellular lipoprotein hydrolysis, and/or oxidative or enzymatic modification of lipoproteins [54]. The presence of membranous debris seen on histopathologic examination corresponds to large drusen and severe RPE pigmentary changes in the central macula that are seen on clinical examination [53, 55–58]. The Curcio lab has found that much of the membranous debris is actually lipoprotein particles [55]. 

On TEM, the lipid particles also appear as “electron lucent” 75 nm diameter round particles within BrM including BlamD, BlinD, and drusen [59]. In the earliest BlinD lesions, the electron lucent particles form a line within the inner collagenous layer adjacent to the RPE basement membrane [58–60]. Using lipid-sparing OTAP TEM, Curcio et al. observed that these electron lucent particles are in actuality, solid, 80 to 100 nm diameter particles that occupy >30% of the inner collagenous layer in normal eyes from patients older than 60 years [59]. Using quick-freeze/deep-etch electron microscopy, Ruberti et al. also identified lipid particles that resemble lipoproteins, forming a thin, densely packed layer external to the RPE basal lamina in old, normal eyes, and theorized that these lipid particles may eventually lead to a confluent lipid wall capable of isolating the outer retina from its choroidal blood supply [61]. The volume fraction and mass occupied by these lipoprotein particles were found to increase monotonically with age in both the inner collagenous layer and elastic layer, but not in the outer collagenous layer [62]. Importantly, the quantity of lipoprotein-associated lipids found in BrM accounts for a large portion of the accumulated lipids measured in this tissue [62]. The pattern of lipoprotein particle accumulation to the inner portion of Bruch's membrane has been theorized to originate potentially from the RPE rather than systemic delivery via the choriocapillaris, as will be discussed below.

## 5. The Lipid Composition of Aging Bruch's Membrane and Drusen

Recent work suggests that the lipoprotein particles within Bruch's membrane are distinct from plasma lipoproteins and have been found to contain free and esterified cholesterol [63], phosphatidylcholine (PC), and apolipoprotein B100 [64]. Curcio et al. found that esterified cholesterol comprised 60% of total cholesterol within these lipoproteins, and esterified cholesterol was 7-fold higher in the macula than periphery [59]. The esterified cholesterol within BrM was 16- to 40-fold-enriched relative to plasma [59]. If the extracellular deposits of lipid were derived from blood, more than 90% of phospholipid would be phosphatidylcholine. In actuality, these lipoproteins are comprised of no more than 50% phosphatidylcholine [59]. These compositional differences suggest that these particles are distinct lipoproteins from those in the plasma [59]. 

BrM lipoproteins are rich in the fatty acid linoleate and poor in docosahexaenoate and contain unesterified cholesterol at higher concentration than in the neurosensory retina. Their density fraction contains apolipoproteins B100, A-I, and E [64, 65]. As photoreceptor outer segments are rich in docosohexanoate, the lack of this fatty acid in lipoproteins suggests that there is an intermediary process within RPE cells where fatty acids are repackaged for neutral lipid secretion [57]. These findings indicate that the lipoproteins in Bruch's membrane cannot be derived directly from photoreceptors without considerable intracellular processing by the RPE, additional cholesterol sources, or both. Enzymatic colorimetry, fluorometry, electrospray ionization mass spectrometry (ESI/MS), and negative stain electron microscopy showed that Bruch's membrane lipoproteins are eluted in two peaks. Peak 1 (plasma LDL-HDL density range) lipoproteins contain predominantly phospholipid and unesterified cholesterol, which morphologically appeared heterogeneous. Peak 2 (plasma VLDL density range) lipoproteins were enriched with esterified cholesterol and were approximately 100 nm diameter round electron-lucent particles. Based on these data, Li et al. concluded that BrM lipoproteins do not resemble plasma lipoproteins in density profile, cholesterol distribution, or morphology [65]. 

Recent studies of drusen composition and substructure have helped us to understand how these lesions form. Drusen contains neutral lipids, with long chain fatty acid cholesteryl esters and nonesterified cholesterol constituting ~3.2% of the dry weight [60, 65, 66] and can occupy up to 37–44% of drusen volume [64]. Approximately, 30% of drusen exhibit at their bases, 15-**μ**m diameter cores that are enriched in nonesterified cholesterol and nonfibrillar amyloid [13]. Drusen also contains at least 29 different proteins, including apolipoproteins (e.g., apoE, apoB) [67, 68]. Other major proteins include vitronectin, complement component 9, clusterin, and scavenger receptor B2 [35, 69]. These components suggest that the innate immune response is involved in drusen formation.

## 6. Consequences of Lipid Accumulation in Bruch's Membrane

While we know that some of the membranous debris that Sarks et al. [53] first characterized is lipoproteins, others have speculated that membranous debris is membrane-bound packets of RPE cytoplasm released by the RPE [67], RPE basal infoldings disintegrating with age [70], Muller cell processes invading BlamD [55], or subcellular blebs after sublethal cytotoxicity [71]. A 3-phase model of basal deposit formation has been proposed which is based on progressive RPE cell injury with early secretion of basement membrane like material, polymerization or condensation of material to produce long spacing collagen and dense amorphous material, and finally, release of membranous debris that comprises BlinD and large drusen [51]. 

In addition to the release of intracellular material, the accumulation of lipoprotein particles in the inner collagenous layer with chronological aging, and their presence within drusen, basal laminar, and linear deposits suggests that their deposition is a critical antecedent event in the formation of these histopathologic markers of AMD. AMD lesion formation has thus been conceptualized as sharing mechanisms with atherosclerotic plaque formation, where LDL retention within the arterial wall initiates a cascade of pathologic events called the “response to retention” hypothesis [13]. In atherosclerosis, apolipoprotein B100 lipoproteins, due to a number of changes to the subendothelial matrix, become trapped and then oxidatively modified. These modifications stimulate different biological processes including innate immune system-mediated inflammation, which induces a cascade of pathologic events that culminate in atherosclerotic plaques [72]. In AMD, the following evidence supports the “response to retention” hypothesis: (1) apoB100-containing lipoproteins accumulate in BrM in the same location as basal deposits and drusen, (2) oxidatively modified proteins and lipids are present in BrM; our laboratory has shown that oxidized apoB100 lipoproteins accumulate in BrM including drusen and basal deposits in AMD and that oxidized lipoproteins induce a pathologic phenotype to RPE cells [73], and (3) the accumulation of inflammatory mediators within drusen and basal deposits indicates a role for the innate immune response [50].

## 7. Consequence of Oxidized Lipids

Oxidation of retained apoB100 lipoproteins is critical to the development of atherosclerosis [72], and there is emerging evidence for oxidized lipoproteins in AMD. Oxidized lipoproteins can trigger complement activation [74]. CD36 is the principal receptor implicated in uptake of oxidized low-density lipoproteins and is expressed by the RPE. CD36 ligands also promote sterile inflammation through assembly of a Toll-like receptor 4 and 6 heterodimer [75]. Uptake of oxidized LDL by the RPE is CD36 dependent and appears to bind to oxidized PAPC (1-palmitoyl-2-arachidonoyl-sn-glycero-3-phosphocholine) and PLPC (1-palmitoyl-2-linoleoyl-sn-glycero-3-phosphocholine) that are contained in oxidized lipoproteins [76–78]. It appears therefore that CD36 may have a role not only in the clearance of oxidized lipids from BrM [79], but also in subsequently inducing an immune response [75].

One pathogenic component of oxidized lipoproteins is oxysterols, which are metabolites of unesterified cholesterol that are generated in vivo either as a byproduct of mitochondrial enzymes or from LDL oxidation. In atherosclerosis, oxysterols contribute to the conversion of macrophages into foam cells [80]. The human eye is now known to produce significant quantities of 7-ketocholesterol and related substances as a direct result of photoreceptor function. Much of the oxysterols (7-ketocholesterol, 24-hydroxycholesterol, 25-hydroxycholesterol, 27-hydroxycholesterol) are generated from the oxidative conversion of cholesteryl esters. Oxysterols have cytotoxic and proinflammatory properties on RPE cells. For example, Dasari et al. have recently shown that 27-hydroxycholesterol increases reactive oxygen species generation, glutathione depletion, increased ER stress, reduced mitochondrial membrane potential, Ca2^+^ dyshomeostasis, inflammation through activation of nuclear factor *κ*B (NF*κ*B), A*β* peptide production, and eventually apoptotic-mediated cell death in cultured RPE cells [81]. Oxysterols induce IL-8 production and secretion [82]. In addition, RPE cells are able to uptake oxysterols at a high rate. This raises the possibility that ingestion of sufficient concentrations of oxysterols by RPE cells can induce an inflammatory response or induce apoptosis. To date, while no human histopathologic study has identified oxysterols in Bruch's membrane or drusen, 7-ketocholesterol has recently been found to be localized in deposits within the choriocapillaris and Bruch's membrane of aging monkeys [83]. Given the aging-associated accumulation of cholesteryl esters in drusen and Bruch's membrane as described above, and their tendency to become oxidized into oxysterols, the toxic role of 7-ketocholesterol and/or the failure to neutralize oxysterols may explain in part how oxidized lipoproteins play a role in AMD and points towards a novel therapeutic target [84].

## 8. The Origin of Lipoproteins

Since lipoproteins are synthesized in the liver, the plasma has been implicated as the source of lipoproteins which accumulate in Bruch's membrane [59, 85]. The apoB100 backbone and size of these particles suggest that they were most closely related to LDL or VLDLs. This has prompted a search for an association between serum lipid and AMD.

### 8.1. AMD and Systemic Lipid

To date, epidemiological studies on the association between serum lipid and AMD risk have been inconsistent [86–94]. Part of this variability may have resulted from reporting different forms and stages of AMD with different lipid profiles. For example, several studies have not found an association between serum lipid profile and AMD risk [86, 88, 91]. Elevated HDL but not total cholesterol was associated with an increased risk of AMD in a study by Van Leeuwen et al. [86]. Nonneovascular AMD was unrelated to cholesterol level in a study by Hyman et al. [88]. No difference in total cholesterol, triglycerides, phospholipids, high- and low-density lipoprotein-cholesterol (HDL-C and LDL-C) concentrations were observed between AMD patients and controls in the study by Abalain et al. [91], and there was no significant difference in the concentration of the Lipoprotein(a) between the AMD and control groups in the work by Nowak et al. [92].

Some correlations between serum lipid and AMD have been made when investigators have looked specifically at high-density lipoproteins. Again, the results have been conflicting. The Rotterdam [86] and Pathologies Oculaires Liees al'Age [95] studies found an association between AMD risk and high-density lipoprotein-cholesterol (HDL-C). When separating by disease type, Klein et al. [87] showed that higher serum HDL-C at baseline was associated with pure geographic atrophy or advanced non-neovascular AMD (RR per 10 mg/dL HDL-C, 1.29; 95% CI, 1.05–1.58; *P* = 0.01), while Hyman et al. [88] revealed a positive association between HDL level and neovascular, but not non-neovascular AMD (OR = 2.3), and dietary cholesterol level (OR = 2.2). 

On the other hand, different studies have found an inverse correlation with HDL and AMD risk. Reynolds et al. [96] recently demonstrated that elevated HDL is associated with a reduced risk of advanced AMD, especially the neovascular (NV) subtype (*P* value <0.05, 0.03, resp.), and that higher low density lipoprotein (LDL) is associated with increased risk of advanced AMD and the NV subtype (*P* value <0.03, 0.04, resp.). When looking for an association of serum lipids with advanced AMD in multivariate modeling, Reynolds et al. also found significant trends with a higher quartile of LDL and increasing AMD risk (*P* for trend <0.03, 0.01). Higher total cholesterol was also associated with AMD risk when controlling for all covariates and genotypes. 

These findings were compatible to findings in previous large studies, such as the Blue Mountain Eye Study (BRMES), a population based cohort study, and the Eye Disease Case Control Study (EDCCS). The BRMES found that increased HDL cholesterol was inversely related to incident late AMD (RR per standard deviation (SD) increase, 0.74; 95% CI, 0.56–0.99). Elevated total/HDL cholesterol ratio predicted late AMD (RR per SD increase, 1.35; 95% CI, 1.07–1.70) and geographic atrophy (RR per SD, 1.63; 95% CI, 1.18–2.25) [97]. The EDCCS also reported a statistically significant 4-fold increased risk of exudative AMD with the highest serum cholesterol levels [93].

The association between the use of cholesterol-lowering medications, including statins, and AMD has also been intensively studied. Like the systemic lipid studies, these results have also been inconsistent [98–105]. The Rotterdam and several other studies did not find a relationship between cholesterol-lowering medication [98–100] including statins [101] and risk of AMD. McGwin Jr. et al. [102] evaluated the association between the use of cholesterol-lowering medications and AMD in the Atherosclerosis Risk in Communities (ARIC) study, a large, prospective, population-based cohort study conducted in four communities across the United States. The large sample size in this study was a strength over previous research on statins and AMD risk. Adjusting for the confounding influence of age, gender, and race, the authors found a significant relationship between AMD and use of cholesterol-lowering medications (OR, 0.79; 95% CI, 0.63–0.99) [106].

### 8.2. AMD and Cardiovascular Diseases

Some studies have suggested an association between AMD and clinical manifestations of cardiovascular disease (CVD). For example, results obtained from a cross-sectional, large population-based study in The Netherlands showed a 4.5-fold increased risk of late AMD associated with atherosclerotic lesions at the carotid bifurcation [16, 107]. Cardiovascular disease and AMD share some of the same risk factors, including high body mass index (BMI), elevated C-reactive protein and other cytokines, and smoking history [16, 20, 21, 108–110]. A study of 930 individuals found that serum C-reactive protein was significantly elevated in individuals with advanced AMD [16, 111]. An epidemiologic correlation between atherosclerosis and AMD has also been established [94, 97]. It is possible that cardiovascular disease could provide a comparison model for the role of cholesterol in AMD pathogenesis [59]. The association of cardiovascular disease with AMD, similar risk factors in both and evidence of lipoprotein deposits in both conditions favors the response to retention hypothesis in AMD.

### 8.3. Evidence for Local Production of Lipoproteins

BrM lipoproteins do not resemble plasma lipoproteins in density profile, cholesterol distribution, and morphology. The transcripts for apoB100, apoA-I, apoE, microsomal triglyceride transfer protein, and other genes involved in lipoprotein synthesis are expressed by the RPE [69, 112–114]. RPE cell lines in vitro secrete lipoprotein particles [112, 115]. While the in vitro environment may not replicate the in vivo conditions, these observations have raised the possibility that the RPE assembles and secretes a large, possibly novel, lipoprotein particle, and that the lipoproteins which accumulate in Bruch's membrane are locally rather than systemically derived. Why would the RPE secrete lipoproteins? Cardiac myocytes are known to secrete lipoproteins as a protective mechanism against the toxic accumulation of lipid [116]. Large oil red O-binding intracellular droplets have been reported in the RPE with aging which could stimulate the synthesis and secretion of lipoproteins [115]. An obvious potential source, not present in other cell types for lipid accumulation, is the constant recycling of POS tips by the RPE. In fact, as mentioned above, the RPE cycles approximately 30,000 lipid rich POS tips per day [28]. The excess lipid, such as phospholipids and unesterified cholesterol derived from POS, after being internalized within the RPE could therefore serve as a source for lipoprotein formation. The apparent dissimilarity in lipid composition between BrM lipoproteins and POS tips can be reconciled by a complex intracellular lipid processing by the RPE where certain elements, such as docosahexaenoate are recycled to the POS, and other lipids are instead packaged into lipoproteins for secretion in the Bruch's membrane for systemic elimination via the choriocapillaris [117]. The RPE contains lipoprotein receptors and thus internalize lipoproteins [118–120]. Curcio et al. have postulated that the BrM lipoprotein is a consequence of the RPE eliminating remnants of lipoprotein components after the RPE has extracted specific nutrients (e.g., xanthophylls, cholesterol, and vitamin E) for use by photoreceptors. According to this model, cholesteryl ester-rich lipoproteins from plasma are taken up by RPE, stripped of nutrients, and repackaged for secretion in BrM as large, cholesteryl ester-rich apoB-lipoproteins [13].

This theory does not eliminate the possibility that lipoproteins that accumulate in Bruch's membrane are of hepatic or intestinal origin arriving from the plasma, especially because lipoproteins are delivered to the retina for nutrition. The evidence against systemic delivery of lipoproteins in Bruch's membrane at this time is based primarily on the distinct differences in size and lipid composition. The variable size, however, could be the result of lipoprotein aggregation or enzymatic processing. Likewise, the difference in lipid composition could be the result of local enzymatic processing after lipoproteins become retained in Bruch's membrane. Identification of the relative contribution by the RPE and the systemic circulation would be helpful in devising treatments that target reducing lipoprotein accumulation to reduce drusen formation.

## 9. AMD and Lipid-Related Genes

Recently, genetic information has provided novel insights into the development of AMD. The most replicated linkage signals have been identified at 1q25-31 and 10q26 [16, 121]. These studies have also identified polymorphisms in complement factor H, C3, HTRA1, ARMS2, and mitochondrial DNA polymorphism A4917G as risk factors, and complement factor B and apolipoprotein E4 (APOE4) as protective factors [122–128]. Just as the discovery of complement factor H has provided an unexpected role for complement-mediated inflammation in the development of AMD [125, 129–131], recently identified genetic links of lipid related genes may provide further insights into how lipid biology influences AMD.

Several genome-wide scans have been published recently, in which several lipid-related genes have been associated with AMD risk. The gene of apolipoprotein E (ApoE) encodes for a plasma protein participating in the metabolism of cholesterol and other lipids [132], and since it is found in drusen [133, 134], it has been evaluated genetically for an association with AMD risk. Apo E2 (Cys 112, Cys 158), Apo E3 (Cys 112, Arg 158), and Apo E4 (Arg 112, Arg 158) are encoded by three alleles: epsilon 2, epsilon 3, and epsilon 4, respectively, which are located on chromosome19 [132]. These three main isoforms are known to differ in one amino acid at one of the two primary protein sites, which alters their function. While some studies show little association of apoE and AMD risk [135], other well-conducted studies do provide evidence for an association with AMD. Most studies confirm that the ApoE *ε*4 allele is a protective variant of the gene that diminishes the risk for developing AMD [136] and its late form [127] and also reduces the risk of developing AMD in individuals with a family history [137]. Subjects who had at least one epsilon 4 allele had a higher macular pigment optical density across the macula than subjects without this allele, which may provide protection against photo-oxidative damage [138]. Meanwhile the *ε*2 allele, on the contrary, has been linked with the risk of developing AMD [127, 139]. The frequencies of the E2 and E4 alleles in Caucasians are approximately 8% and 15%, respectively. Allele- and genotype-based tests of association have indicated a risk effect of up to 20% for E2 and a protective effect of up to 40% for E4. E2 appears to act in a recessive mode and E4 in a dominant model [136]. Souied et al. have offered two different hypotheses explaining the protective mechanism of Apo E4 in the development of AMD. Apo E4, in contrast to Apo E2 and 3, does not contain disulfide bridges; therefore, being smaller, it may be more effectively transported through Bruch's membrane. Alternatively, apo E4 is positively charged while E2 and E3 are negatively charged. This difference in charge may contribute to differences in their ability to clear debris through Bruch's membrane [127]. 

Ironically, the Apo E4 allele is associated with hypercholesterolemia and greater risk of ischemic heart disease, whereas the Apo E2 allele is considered a protective factor against the development of this disorder [127]. The ApoE *ε*4 allele is associated with increased risk and earlier onset of Alzheimer disease (AD), and conversely the ApoE2 allele is protective against AD [139]. 

The effect of ApoE on AMD risk is not consistent with findings in AD and ischemic heart disease. However, the capacity of Apo E to mediate cholesterol efflux from cells suggests that its function as a cholesterol carrier protein may be involved in AMD. Variability in the efficacy of cholesterol redistribution may account for the association of Apo E with AMD. Moreover, Apo E is an A*β*-binding protein and may thereby be involved in A*β* aggregation and clearance, which may account for the specific association with AMD. Finally, C-terminal truncated toxic fragments of ApoE can enter the cytosol and exert detrimental effects on the cytoskeleton and mitochondria which may lead to cell death [140]. 

Curcio el al. [13] suggested the following scenario as a possible way in which apoE might have different effects in AMD and CVD. In atherosclerosis, cholesterol efflux into the intima prevents macrophages from differentiating into foam cells, and apoE4 hinders this efflux, thus exacerbating atherosclerosis. In AMD, cholesterol exported from the RPE within lipoproteins accumulates in BrM and promotes lesion formation. Perhaps the opposing effect of cellular cholesterol export into BrM and the vascular intima helps to explain the opposite direction of apoE4's influence in AMD and CAD. This theory presumes that a significant source of Bruch's membrane lipid originates from the RPE.

Hepatic lipase (LIPC), a gene located on chromosome 15q22, was recently discovered to be associated with AMD in a large, genome-wide association study by Neale et al. [141]. LIPC encodes hepatic triglyceride lipase, which catalyzes the hydrolysis of phospholipids, monoglycerides, diglycerides, triglycerides, and acyl-CoA thioesters and is a critical enzyme in HDL metabolism. Hepatic lipase also binds heparin and has the dual functions of a triglyceride hydrolase and a ligand/bridging factor for receptor-mediated lipoprotein uptake. This genetic finding has been replicated in another genome-wide association study by Chen et al. [142], who showed strong association signals with alleles at two loci (LIPC, *P* = 1.3 × 10^−7^; CETP, *P* = 7.4 × 10^−7^) that were previously associated with high-density lipoprotein cholesterol (HDL-C) levels in blood. They also observed an association with AMD and HDL-C-associated alleles near lipoprotein lipase (LPL; *P* = 3.0 × 10^−3^) and ATP-binding cassette (ABCA1; *P* = 5.6 × 10^−4^) [142].

Reynolds et al. [96] explored the relationship between the LIPC genotype and serum lipids. The mean HDL level increased with each T allele of the LIPC gene (*P* < 0.05 for trend). Total cholesterol, LDL, and triglycerides were not significantly associated with LIPC. Models controlling for age and other covariates not including HDL showed a protective effect of LIPC on AMD. Their results showed that the TT genotype of the LIPC gene was associated with a reduced risk of both neovascular and geographic atrophy subtypes of advanced AMD [96]. There was a trend for increasing HDL with increasing LIPC T alleles. However, HDL level did not seem to mediate the association between LIPC and AMD based on the independent associations of both LIPC and HDL when considered simultaneously [96]. Thus, at present, while a genetic link for LIPC and AMD has been established, the present epidemiologic evidence suggests that LIPC's effect is not mediated through plasma HDLs. In fact, Neale et al. postulated that the LIPC association may not be the result of an effect on HDL levels, but it could represent a pleiotropic effect of the same functional component [141]. Since LIPC is expressed in the retina, an undefined local effect by LIPC that protects against AMD is possible [141]. Future studies determining the role of LIPC in the eye may provide insight into its contribution to AMD pathology. 

Several other genes that have a role in lipid metabolism have been reported to have an association with AMD. For example, the ABC transporters play pivotal roles in cellular lipid transport, and mutations in ABCA-transporter genes have been shown to result in hereditary diseases involving major physiological processes in the cardiovascular, respiratory, visual, and integumentary systems. Accumulated evidence also suggests that ABCA-transporters play critical roles in the pathogenesis of complex multifactorial disorders with a high incidence such as atherosclerosis, AMD, and Alzheimer's disease [143]. Expression of reverse cholesterol transport proteins ATP-binding cassette A1 (ABCA1) and scavenger receptor BI (SR-BI) in the retina and retinal pigmented epithelium has been reported. RPE cells may utilize reverse cholesterol transport (RCT) activity to move lipid into BrM, mediated through ABCA1 and SR-BI [144]. A polymorphism in Elongation of very long chain fatty acids-like 4 (ELOVL4), which is involved in fatty acid biosynthesis, is also associated with AMD [145], but the role of this gene in AMD has not been confirmed in other studies [146].

In a study conducted in French and north American AMD patients and control group who did not carry the prevalent complement factor H (CFH) nor age-related maculopathy susceptibility 2 (ARMS2) polymorphisms, an association between AMD and the scavenger receptor class B member 1 (SCARB1) gene was seen among the study subjects. SNP rs5888 of the SCARB1 gene codes for SRBI, which is involved in the lipid and lutein pathways. The genotypic distribution of the rs5888 polymorphism was significantly different between cases and controls in the French population (*P* < 0.006). Heterozygosity at the rs5888 SNP increased risk of AMD compared to the CC genotypes [147]. SRB1, a multiligand cell surface receptor, is known to mediate selective cholesterol uptake and cholesterol efflux. Furthermore, recent studies have shown that SCARB1 is also involved in uptake of vitamin E and lutein giving further support to a possible role of *SCARB1* in AMD.

## 10. Conclusions

At present, the above epidemiologic, molecular, and cell biology, and genetic studies have not integrated the findings into a unified explanation for the role of lipids and lipoproteins in AMD. To establish a unified role for lipids in AMD, future studies should utilize the following evidence in their design, as outlined in this paper: (1) lipoproteins, which have a composition that is distinct from plasma lipoproteins, accumulate in Bruch's membrane with aging before hallmark Bruch's membrane AMD lesions. (2) Oxidized lipoproteins have been identified in Bruch's membrane, basal deposits, and drusen of AMD specimens, suggesting a possible trigger for the induction of inflammation and drusen formation. (3) Multiple epidemiologic studies suggest no substantive role of plasma lipoproteins in AMD, and therefore reduce the likelihood of plasma lipoproteins as a source of Bruch's membrane lipoprotein accumulation. (4) Cell biology and molecular pathology studies suggest a role for local that is RPE synthesis and secretion of apoB100 lipoproteins as a plausible explanation for their origin in Bruch's membrane. The definitive study that would determine the origin of Bruch's membrane lipoproteins would be to topographically knock out lipoprotein production in the liver, the eye, both, and neither, to determine the relative contribution of lipoprotein accumulation in Bruch's membrane under these experimental conditions. Given the availability of genetically altered mice related to lipoprotein metabolism and Cre recombinase technology to topographically knock out a gene of interest, this experiment is feasible and is, in fact, ongoing in our laboratory. (5) Genetic variations have been identified as increasing risk for AMD. Establishing mechanistic evidence of these lipid-related genes with aspects of an AMD phenotype in animal models will define their biologic contribution. Alternatively, the epidemiologists could, in future studies, analyze the role of plasma lipids in the context of genotype before eliminating the role of systemic lipids in AMD. This approach may not identify systemic lipids as a major risk factor in AMD, but it might identify susceptibility in a subset of patients who are at risk for AMD because of genotype and plasma lipid levels. While lipoproteins in Bruch's membrane both accumulate and can become oxidized, experimental proof of establishing how lipoprotein accumulation in Bruch's membrane leads to RPE cell death or drusen formation is currently lacking and would establish a definitive role for lipids in AMD. In the future, it will be necessary for the epidemiologists, genetics, and molecular and cellular biologists to work together to definitively determine how lipid biology influences the development of AMD. It is hoped that greater understanding of the molecular biology of AMD lesions will provide a knowledge based on which to develop multiple treatment targets for the benefit of patients with AMD.
